# *Leishmania tarentolae*: a vaccine platform to target dendritic cells and a surrogate pathogen for next generation vaccine research in leishmaniases and viral infections

**DOI:** 10.1186/s13071-023-05651-1

**Published:** 2023-01-26

**Authors:** Claudio Bandi, Jairo Alfonso Mendoza-Roldan, Domenico Otranto, Alessandro Alvaro, Viviane Noll Louzada-Flores, Massimo Pajoro, Ilaria Varotto-Boccazzi, Matteo Brilli, Alessandro Manenti, Emanuele Montomoli, Gianvincenzo Zuccotti, Sara Epis

**Affiliations:** 1 Department of Biosciences, Pediatric CRC “Romeo ed Enrica Invernizzi”–University of Milan, Milan, Italy; 2 Department of Veterinary Medicine, University of Bari, Valenzano, Italy; 3 VisMederi, Siena, Italy; 4 Department of Molecular and Developmental Medicine, University of Siena, Siena, Italy; 5 Department of Biomedical and Clinical Sciences, Pediatric CRC “Romeo ed Enrica Invernizzi”–University of Milan, Milan, Italy; 6 Department of Pediatrics, Ospedale dei Bambini-Buzzi, Milan, Italy

**Keywords:** Protozoa, Leishmaniasis, Antigens, Parasites, Viral vaccines, Immunization

## Abstract

Parasites of the genus *Leishmania* are unusual unicellular microorganisms in that they are characterized by the capability to subvert in their favor the immune response of mammalian phagocytes, including dendritic cells. Thus, in overt leishmaniasis, dendritic cells and macrophages are converted into a niche for *Leishmania* spp. in which the parasite, rather than being inactivated and disassembled, survives and replicates. In addition, *Leishmania* parasites hitchhike onto phagocytic cells, exploiting them as a mode of transport to lymphoid tissues where other phagocytic cells are potentially amenable to parasite colonization. This propensity of *Leishmania* spp. to target dendritic cells has led some researchers to consider the possibility that the non-pathogenic, reptile-associated *Leishmania tarentolae* could be exploited as a vaccine platform and vehicle for the production of antigens from different viruses and for the delivery of the antigens to dendritic cells and lymph nodes. In addition, as *L. tarentolae* can also be regarded as a surrogate of pathogenic *Leishmania* parasites, this parasite of reptiles could possibly be developed into a vaccine against human and canine leishmaniases, exploiting its immunological cross-reactivity with other *Leishmania* species, or, after its engineering, for the expression of antigens from pathogenic species. In this article we review published studies on the use of *L. tarentolae* as a vaccine platform and vehicle, mainly in the areas of leishmaniases and viral infections. In addition, a short summary of available knowledge on the biology of *L. tarentolae* is presented, together with information on the use of this microorganism as a micro-factory to produce antigens suitable for the serodiagnosis of viral and parasitic infections.

**Graphical Abstract:**

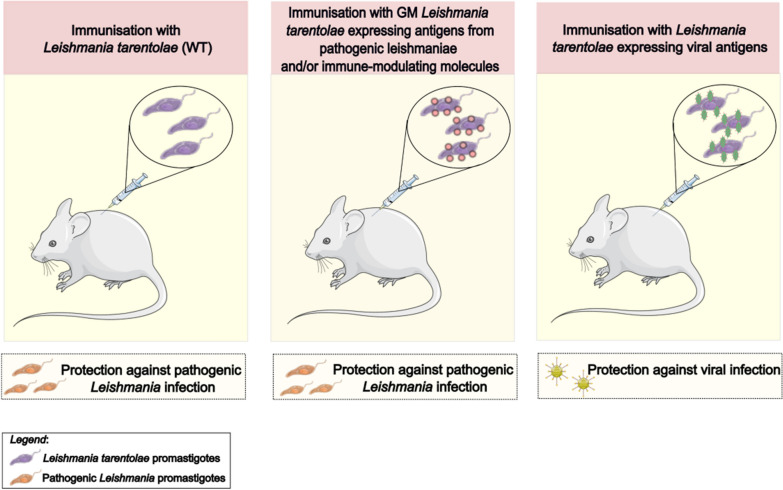

## Background

### Dendritic cells, antigen delivery and *Leishmania tarentolae*

**Fig. 1:**
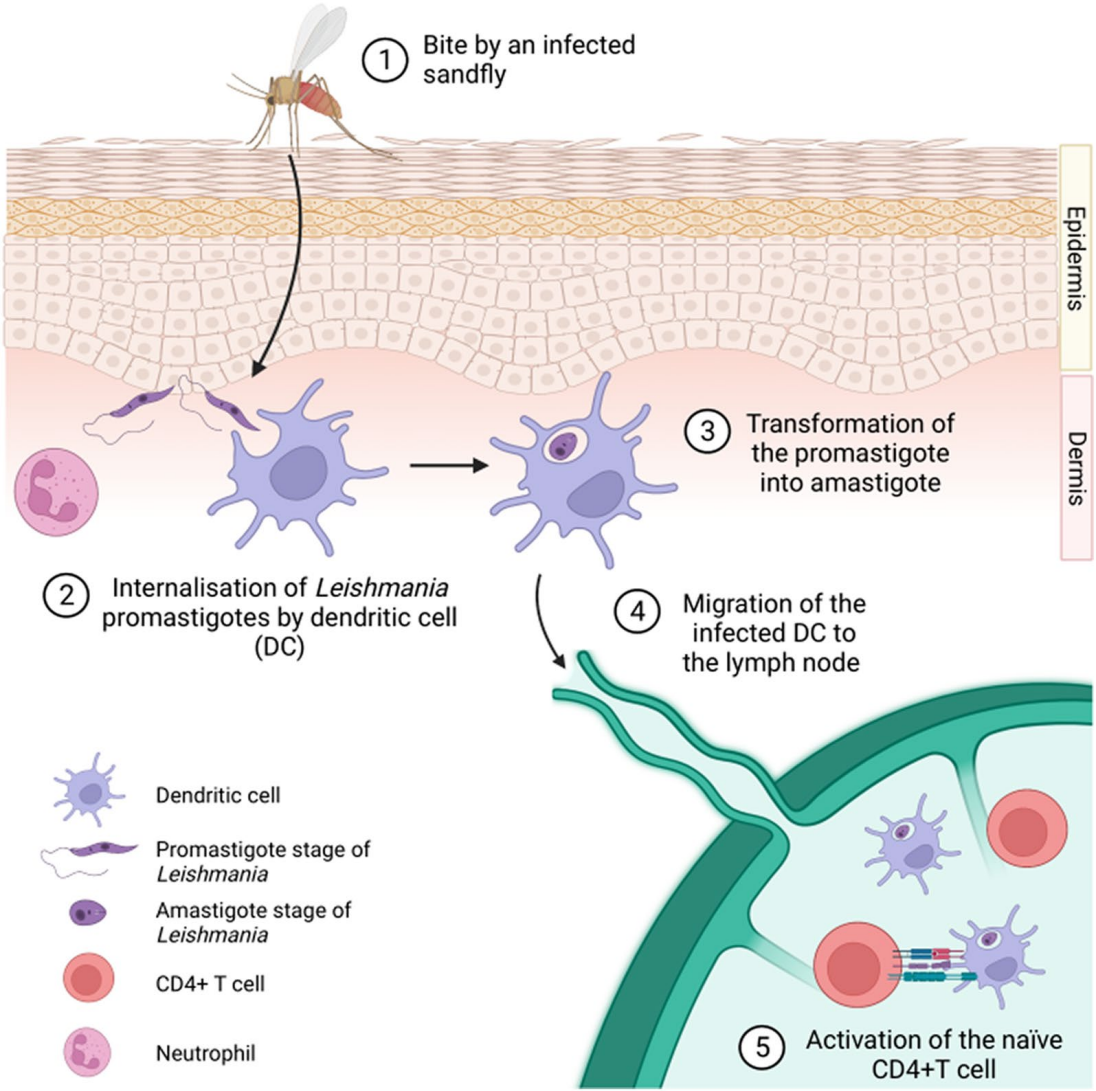
Schematic representation of the cutis of a mammal and a lymph node. A *Leishmania*-infected Phlebotominae sand fly is also represented (1). A dendritic cell (DC) is represented during the process of phagocytosis of a *Leishmania* promastigote (*2*), the transformation of the promastigote into the amastigote stage (*3*) and then the migration of the infected DC towards the lymph node (*4*). Inside the lymph node, DCs present the antigens to CD4+ T cells. A neutrophil is also shown as these cells play a role in the first phases of mammalian infection by *Leishmania* spp (*5*)

Upon their invasion of vertebrate tissues, infectious microorganisms are typically exposed to the action of phagocytic cells, including dendritic cells (DCs). DCs subsequently transport the engulfed microbes to lymph nodes for processing of the microbial proteins into peptides and for the presentation of peptides to CD4+ T cells. DCs thus act as professional antigen presenting cells (APCs), playing a key role in the initiation of the adaptive immune response [1]. Compared to most pathogens, *Leishmania* parasites are rather unusual in that they exploit APCs of the myeloid cell line as a favourable habitat for their survival and reproduction [2]. In other words, *Leishmania* parasites are not only passively exposed to the phagocytic activity of DCs and macrophages but they specifically target phagocytic cells thanks to specific surface receptors. Once engulfed by DCs and macrophages, *Leishmania* parasites subvert the activation of these cells, inhibiting their oxidative and lytic antimicrobial responses, and use these cells as a mode of transport to the lymph nodes [3]. In summary, *Leishmania* parasites display one of the desired requirements for a vaccine vehicle: a specific targeting of DCs and lymph nodes (Fig. 1).

Despite this feature, however, candidate vaccines against leishmaniases based on the use of whole *Leishmania* cells, either inactivated or attenuated, have not yet been developed into licensed and available vaccines for human or canine use [4–6]. The major drawbacks of inactivated anti-*Leishmania* vaccines can be summarized as follows: (i) the limited immunogenicity of inactivated vaccines, as compared to attenuated ones; (ii) an improper modulation of the immune response, associated with the Th2-biasing properties of pathogenic *Leishmania* species; and (iii) the limits in the scalability to mass production of inactivated cells from pathogenic *Leishmania* species, and the logistical difficulties in the wide-scale distribution of the derived vaccine preparations [7]. These drawbacks, all of which are typical of inactivated vaccines—in particular the property of limited immunogenicity—have been overcome in other preparations using attenuated living microbes as vaccines. However, this latter approach entails possible safety issues [8] and even greater logistical difficulties for large-scale vaccine campaigns. In this context, the generation of gene-deleted clones to be used in vaccination programs can minimize the risk of reversal to virulence by attenuated pathogenic *Leishmania* strains [6].

The limits of inactivated and attenuated vaccines have been circumvented using surrogate pathogens as vaccines. For example, the Bacillus Calmette-Guerin (BCG) vaccine is derived from a cattle strain of the bacterium *Mycobacterium bovis* and developed as a vaccine against human tuberculosis (caused by *Mycobacterium tuberculosis*) after serial passages in vitro to achieve its attenuation [9]. An attractive feature of BCG vaccine is that this bacterium is suitable for genetic manipulation and therefore for the production of heterologous proteins. This led to the generation of BCG strains expressing antigens from other microorganisms, that have been assayed as candidate vaccines against a variety of pathogens unrelated with the mycobacteria [10]. *Leishmania tarentolae*, a parasite infecting reptiles, shares several features with BCG in terms of its potential to be exploited as a surrogate pathogen for use in vaccinations against leishmaniasis [11]. In addition, *L. tarentolae* has the potential to be developed as a vaccine platform of a wider interest, owing to the suitability of this parasite for genetic manipulation and, therefore, the production of heterologous protein antigens [11, 12].

**Fig. 2:**
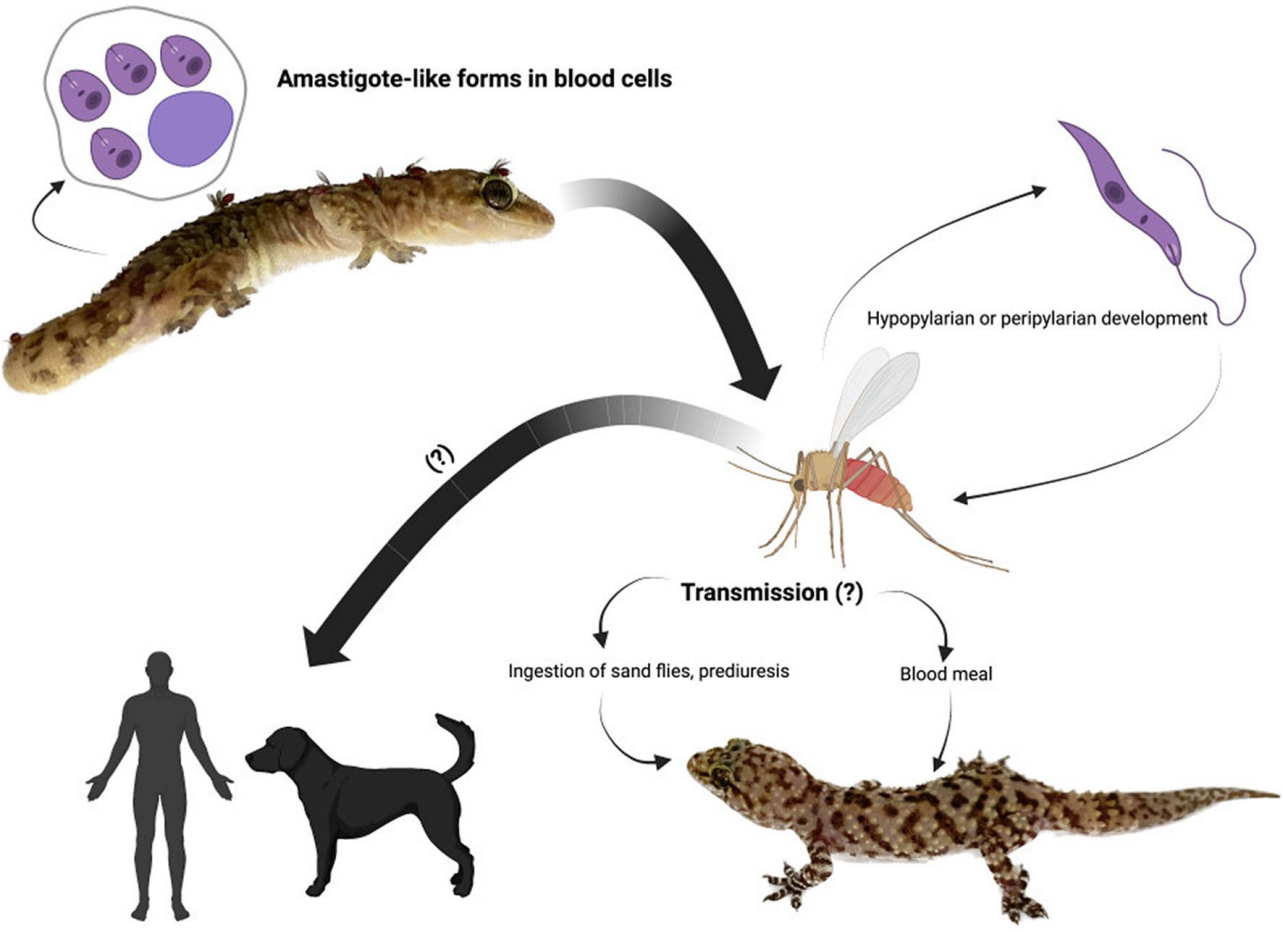
Life-cycle of *Leishmania tarentolae* in sand flies and vertebrate hosts. In reptiles, amastigote-like forms are known to develop in blood cells, but the details of the life-cycle in reptilian hosts are still unknown. Sand flies ingest infected blood cells, and parasites subsequently differentiate into promastigotes, with a hypopylarian or peripylarian type of development. The transmission to vertebrate hosts is likely to occur, in most cases, through the blood meal or through oral ingestion of the fly. Other modes of transmission have also been suggested, including contact with the mucosae of urine droplets containing the parasites. Urine droplets are indeed produced and released by sand fly females during the blood meal through the prediuresis process. Information on transmission and development in mammals is limited

In this article we review current knowledge on this parasite in relation with its potential use in the development of novel types of vaccines, not only against pathogenic *Leishmania* species, but also against a variety of viruses. The use of *L. tarentolae* to produce antigens for serological diagnosis will also be discussed. A schematic representation of the life-cycle of *L. tarentolae* is presented in Fig. 2.

## *Leishmania tarentolae*: natural history and evidence of infectivity in mammalian hosts

Although *L. tarentolae*, the type species of the subgenus *Sauroleishmania* [13], was described more than one century ago in the gecko *Tarentola mauritanica* [14], information on its natural history remains limited [11] with respect to other species within the subgenera *Leishmania* and *Viannia*. Indeed, the 21 species within the subgenus *Sauroleishmania* have historically been overlooked in leishmaniasis research, being associated with reptiles (from the families Agamidae, Gekkonidae, Lacertidae, Scincidae and Varanidae) and generally regarded to be non-infectious to mammals. In addition, *Sauroleishmania* species are transmitted by sand flies of the genus *Sergentomyia*, which have a primarily herpetophilic feeding behavior [15]. The detection of promastigotes of a protist parasite (*Paleoleishmania proterus*) mixed with reptilian nucleated blood cells in the midgut lumen of a *Palaemyia burmitis* female sand fly in mid-Cretaceous amber (approx. 100 MYA) suggested that *Sauroleishmania* evolved in reptiles [16, 17]. However, phylogenetic analyses placing *Sauroleishmania* as the sister group of the subgenus *Leishmania*, after the divergence of *Viannia*, supported the hypothesis that the ancestor of *Sauroleishmania* evolved in mammals and then switched to reptiles [12–18]. The distribution of *L. tarentolae* is limited to the Old World [19] in association with geckoes in Europe, North Africa and the Middle East [20–22]. The tight association between *Sauroleishmania* spp. and reptile-biting *Sergentomyia* sand flies [23–26] was challenged by the detection of mammalian blood in these sand fly species [27, 28]. These results also raised questions about the potential role of *Sergentomyia* sand flies as vectors of *Leishmania infantum*. Meanwhile, individuals of different species of *Phlebotomus* (e.g. *Phlebotomus perfiliewi, P. perniciosus* [29–31]) have been found positive for *L. tarentolae*, most likely as an effect of their opportunistic feeding behavior, which may be linked with several ecological factors and host availability [32]. These observations are further supported by the experimental infection of *Phlebotomus papatasi* [33, 34], *Ph. perniciosus* and *Phlebotomus sergenti* [34] with *Sauroleishmania* spp. On these premises, we can conclude that *Sergentomyia* is not the sole genus of sand flies in which *Sauroleishmania* can develop and that these *Leishmania* protozoan parasites could also circulate in mammals. Moreover, laboratory experiments have shown that New World sand flies, such as *Lutzomyia longipalpis*, are competent as vectors for *L. tarentolae* [35], thus suggesting the potential circulation of this parasite also in the Americas.

In the sand fly vector, *Sauroleishmania* has traditionally been classified as an hypopylarian microorganism since it develops mainly in the hindgut [36]. However, the development of some *Sauroleishmania* species in the midgut [33, 34, 37] of *Ph. papatasi* and *Ph. perniciosus* (i.e. peripylarian or suprapylarian), but not in *Ph. sergenti*, suggests that *Sauroleishmania* development is influenced by the insect species [34]. In particular, the presence of *L. tarentolae* promastigotes in the stomodeal valve of some *Phlebotomus* species [33, 34] supports the possibility of transmission through the blood meal, whereas the localization in the Malpighian tubules suggests transmission through urine droplets (i.e. the product of prediuresis), bite wounds or contact with the mucosae of the hosts. In addition to the many aspects of the *Sauroleishmania*—sand fly interactions, the potential infectivity of *L. tarentolae* for mammals has recently been supported after the molecular detection of DNA from this parasite in a 300-year-old Brazilian mummy [38]. This discovery also raised some questions about the host range and geographical distribution of this species and advocated for specific investigations aimed at defining their ability to infect mammalian hosts. It is interesting to note that another species belonging to the subgenus *Sauroleishmania*, *Leishmania adleri*, while primarily associated with ectothermic hosts, may infect humans causing transient skin lesions, similar to those observed in cutaneous leishmaniasis [39], and has also been detected in asymptomatic hamsters and mice [40]. Additionally, under laboratory conditions, *L. tarentolae* promastigotes differentiate into an amastigote-like form after uptake by mammalian DCs and macrophages [41–44], hinting at the possibility of natural infection in mammals. In Mediterranean countries, human and canine leishmaniases are endemic, and *L. tarentolae* has been isolated from different species of reptiles [12, 45, 46] and sand flies [28], and its DNA has also been detected by molecular methods in mammals [29, 38, 46, 47]. A recent molecular screening revealed the presence of *L. tarentolae* DNA in humans and sand flies from central Italy and Linosa island [29, 47]. Importantly, infection by *L. tarentolae* has also been hypothesized as being associated with a reduction in anti-*L. infantum* antibody titers in dogs seropositive to canine leishmaniasis, but clinically healthy [48]. Therefore, in regions where *L. tarentolae* is highly prevalent in reptile and sand fly populations, the presence of this parasite may represent a factor capable of inhibiting the development of clinical forms of canine leishmaniasis, which is associated with antibody-mediated inflammatory reactions [11]. This possibility is further supported by the finding of *Sergentomya minuta* as the most abundant sand fly species in canine leishmaniasis endemic areas [31].

 In summary, available data confirm the sympatric circulation of *L. infantum* and *L. tarentolae* in central and southern Italy, also suggesting that geckoes may be infected with *L. infantum*. Furthermore, the interactions of these two species of *Leishmania* in these areas are evidenced in their overlapping circulation in “non-natural” hosts and vectors. Therefore, hosts and vectors that have historically been considered to be non-permissive to the one or the other of the two species could actually play a role in the epidemiological cycle of *L. infantum* and *L. tarentolae*.

## *Leishmania tarentolae* as a biotech tool: use in the production protein antigens for the serodiagnosis of infectious diseases

The classical prokaryotic systems for protein expression and production (e.g. the well-known *Escherichia coli* system) lack essential components for proper protein folding and eukaryotic-type post-translational modifications. Eukaryotic expression systems have thus been proposed in recent decades, although these are characterized by several limitations, such as slow generation time and high costs of culture maintenance [49]. *Leishmania tarentolae* has been proposed as an alternative system for protein production that overcomes these limitations since it combines the advantages of both eukaryotic and prokaryotic systems: the maintenance and growth of *L. tarentolae* is accomplished at a low cost and is easily scalable to industrial production using bioreactors. In addition, *L. tarentolae* boasts a range of post-translational modifications, including mammalian-type *N*-glycosylation [50]. A particular focus of research is a strain of *L. tarentolae* that was originally isolated from lizards and subsequently cultivated in axenic culture for decades. This strain is commercially available as a eukaryotic protein expression platform (In Vivo LEXSY translation system, Jena Bioscience GmbH) and can be used to express both intracellular or secreted proteins under the activity of constitutive or inducible promoters (https://www.jenabioscience.com/). This system has successfully been exploited to produce both antigens for sero-diagnostic applications and vaccine development (see following section), as well as a variety of proteins to be used in crystallography.

A first obvious application of *L. tarentolae* is the production of protein antigens from other trypanosomatids that can be used in serological diagnosis. In 2015 Rooney and co-workers published the first study in which *L. tarentolae* was used to produce antigens from *Trypanosoma brucei*, with the aim to develop a rapid diagnostic test for African trypanosomiases [51]. Another experimental work exploited *L. tarentolae* for the expression of the rK39 antigen of *L. infantum* for serodiagnosis of visceral leishmaniasis (VL); the results showed satisfactory diagnostic accuracy when the antigen was tested on sera from patients with VL [52].

*Leishmania tarentolae* has also been explored as a means to produce protein antigens for the serodiagnosis of viral infections. The gold standard for serological diagnosis of viral infections implies the use of antigens produced in mammalian cells [53], with all the potential limitations, particularly in terms of fast production and rapid application in the case of emerging epidemics from novel pathogens. Two studies have so far been published on the use of protein antigens produced in *L. tarentolae* for the diagnosis of viral infections. In the first study, an antigen from the hepatitis E virus capsid protein, produced in *L. tarentolae*, was successfully used in an enzyme-linked immunosorbent assay (ELISA) for the detection of antiviral antibodies in porcine sera [54]. In a more recent study, antigens from severe acute respiratory syndrome coronavirus 2 (SARS-CoV-2) were thoroughly tested on sera from humans affected by coronavirus disease 2019 (COVID-19) and found to display diagnostic performances comparable to those achieved by antigens produced in human cells [55].

Recombinant protein yield by *L. tarentolae* can be improved in bioreactors and altered culture growth conditions. Pion et al. [56], for example, engineered *Leishmania* for the production of recombinant influenza hemagglutinins in biofermenter culture; changes in the culture volume (2 l) and agitation of the culture led to a tenfold increase in the yield. Taking safety issues into account, the production of recombinant proteins to be used as vaccines or in therapeutic applications should ideally avoid the use of culture media containing components of animal origin [57]. However, promastigotes of *L. tarentolae* are normally cultivated in liquid media with the addition of blood or animal serum, or other components of animal origin [58]. A commonly used medium is brain heart infusion (BHI) growth medium supplemented with animal serum, but there are alternatives for culturing *L. tarentolae*. For example, Fritsche et al. [59] tested yeast extract medium, with hemin as the sole component of animal origin. Schneider's *Drosophila* medium was also successfully tested for cultivation of *Leishmania* spp., but always in combination with animal serum. Finally, a classic study proposed catalase or high concentrations of peroxidase as substitute for hemin in BHI medium [60]. The above efforts to set up optimal media and culture conditions for *L. tarentolae* cultivation will likely increase its suitability as a tool for protein production at the industrial level, a prerequisite for translational application of this microorganism to produce antigens, vaccines or therapeutics [61].

## *Leishmania tarentolae* as a surrogate of pathogenic leishmaniae in terms of vaccination, and the issue of immune modulation in anti-*Leishmania* vaccines

The origin of vaccines is traced back to 1796, when Edward Jenner proposed the inoculation of material from cowpox lesions as a treatment to protect humans from smallpox. In modern terminology, the first vaccine comprised a surrogate pathogen (the cowpox virus) that causes only mild infections in humans but nonetheless elicits cross-protective immunity against the human pathogen, namely the smallpox virus [62]. During the first half of the twentieth century, BCG was developed as a surrogate of the human pathogen *M. tuberculosis* and is still widely used in vaccination campaigns against tuberculosis (see section Background). The use of microorganisms that infect animal species other than humans as surrogates of human pathogens for vaccination targets is thus part of the history of vaccinology and should not be dismissed as an obsolete or useless strategy. Several lines of evidence suggest that *L. tarentolae* could represent a surrogate pathogen and that it is suitable to be assayed as a vaccine against human and canine leishmaniases. First, cross-protective immunity is well documented in *Leishmania* infections (for example see [63] and citations therein). The existence of cross-protective immunity in *Leishmania* species has also been documented at the molecular level [64] or using sub-components of *Leishmania* cells, as in the LBSap vaccine which, while being composed of antigens from *Leishmania braziliensis*, is protective against *Leishmania infantum* [65, 66]. Second, comparative genomics show that *L. tarentolae* shares approximately 90% of its genes with pathogenic species, including *L. infantum* and *Leishmania major*. However, several genes possibly associated with virulence are lacking in *L. tarentolae*, compared to pathogenic *Leishmania* species [67], which is reassuring in relation to safety issues. Third, current evidence indicates that *L. tarentolae* is not pathogenic to mammals, while still being capable of infecting macrophages and DCs, reaching the amastigote state [41, 44, 68]. Finally, the evidence for a circulation of *L. tarentolae* in dogs, and the absence of any evidence for its association with pathological outcomes, further emphasizes the potential utility of this parasite in anti-*Leishmania* vaccination (see [11] and section on *L. tarentolae* natural history and the evidence of infectivity in mammalian hosts).

A major issue that should be addressed to determine the potential of *L. tarentolae* as a vaccine against pathogenic leishmaniae is the type of immune modulation that this reptile parasite determines in mammals. There is a general consensus that hosts displaying classical macrophage activation (M1 activation), with specific polarization of CD4+ T cells towards the Th1 phenotype, are protected against infection and overt disease with most forms of leishmaniases, in both dogs and humans, while hosts displaying alternative macrophage activation (M2 activation) and a Th2-biased response are more exposed to the risk of developing active *Leishmania* infection and overt disease [69, 70]. For this reason, a central focus in leishmaniasis vaccine research is the identification of adjuvants capable of determining a proper polarization of the immune response towards the M1/Th1 profile [71]. Initial studies, using the murine model, showed that *L. tarentolae* determines Th1-type cytokine production in vivo [41]. However, most of the subsequent studies suggested that elicitation of a Th1 response requires co-administration of the appropriate adjuvants, or the concomitant expression of immune-modulating molecules [72, 73]. However, the expression of M2 markers in human macrophages, after stimulation with *L. tarentolae*, was also observed [74]. A recent in vitro study on human DCs reported moderate production of Th1-type cytokines after stimulation by living promastigotes of *L. tarentolae* and also confirmed the penetration of *L. tarentolae* into DCs and their maturation, with expression of surface markers of activation, including MHC class II and co-stimulatory molecules [44]. The capacity of DCs to engulf *L. tarentolae*, the maturation of these cells after exposure to the parasite and evidence for the production, even though moderate, of Th1-associated cytokines are all coherent with the possibility that *L. tarentolae* possesses some of the characteristics required by an anti-*Leishmania* vaccine.

Despite the above theoretical arguments in favor of the potential utility of *L. tarentolae* as a vaccine against pathogenic *Leishmania* species, only a few studies have investigated the issue experimentally, always in rodent models. In a first seminal study, intraperitoneal immunization with *L. tarentolae* was found to achieve a protective immune response against *Leishmania donovani* in BALB/c mice, associated with a strong Th1 response in the absence of adjuvant [41]; this result was, however, not confirmed by other studies. In that study, the promastigotes of *L. tarentolae* were not engineered for the expression of any antigen from pathogenic *Leishmania* species, nor for any proteins capable of stimulating the immune response, but only for the production of the green fluorescent protein. Live, non-engineered, promastigotes of *L. tarentolae* have also been shown to provide cross-protective immunity against *L. major* [75, 76]. The possibility of exploiting wild-type *L. tarentolae* as a vaccine against dog or human pathogenic *Leishmania* species is thus worthy of further investigations (see section Concluding remarks).

## Engineered *Sauroleishmania* strains as vaccines against pathogenic *Leishmania* species

One possible strategy to increase the immunogenicity of *L. tarentolae* as a vaccine against human and dog leishmaniases is to engineer this microorganism for the expression of antigens from pathogenic *Leishmania* species or of immune-modulating proteins associated with M1/Th1 immune activation. Briefly, in anti-leishmania vaccine studies, *L. tarentolae* has been genetically modified (GM) for the production of the following categories of proteins: (i) antigens from pathogenic *Leishmania* species; (ii) immune-modulating proteins of human origin; and (iii) antigens or immune-modulating proteins from the sand fly saliva. Strains of *L. tarentoale* have also been engineered for the production of both immune-modulating proteins and antigens from pathogenic *Leishmania* species. Studies on GM *L. tarentolae* in anti-*Leishmania* vaccination tests are summarized in Table 1. A brief account, focused on protein categories, is also provided in the following paragraphs.

**Table 1:** Use of *Leishmania tarentolae* as an antigen in vaccination or as a vaccine vehicle, or to produce viral proteins or virus-like particles

Type of expressed molecule	Whole Lt and/or purified protein	Expressed molecule	Target infection/disease	Animal model	Selected references
WT	Whole Lt	**//**	Leishmaniasis	Murine	[75, 76]
GM for: GFP	Whole Lt	GFP to trace *Leishmania tarentolae*	Leishmaniasis	Murine	[41]
GM for: pLs prot	Whole Lt and/or purified protein	pLs A2 antigen pLs A2 antigen + cysteine proteinases A and B (CPA and CPB without C terminal extension) pLs *Leishmania* homolog of the receptor for the activated C kinase (LACK) + pLs kinetoplastida membrane protein-11 (KMP11) *Leishmania infantum* lipophosphoglycan 3 (LPG3) pLs cysteine proteinases A and B (CPA, CPB)	Leishmaniasis	Murine Hamster	[73, 77, 80, 86, 106, 107]
GM for: IM prot	Whole Lt	Interferon-gamma-induced protein 10 (IP-10) or CXCL-10 Human neutrophil peptide-1 (HNP-1) Immunogenic *Phlebotomus papatasi* salivary molecule SP15 (PpSP15) Immunogenic *Ph. papatasi* salivary molecule SP15 (PpSP15) + *Phlebotomus sergentii* salivary molecule SP9 (PsSP9)	Leishmaniasis	Murine	[82, 84, 87, 88]
GM for: IM prot + pLs prot	Whole Lt	pLs kinetoplastida membrane protein-11 (KMP11) + N-terminal domain of heat shock protein GP96 (NT-GP96)	Leishmaniasis	Murine	[108]
GM for: other protozoan protein	Whole Lt and/or purified protein	Fusion of immunodominant epitopes of 5* Toxoplasma* antigens: SAG1-ROP16-GRA12-MIC4-M2AP	Toxoplasmosis	Murine	[99]
GM for: viral protein	Whole Lt and/or purified protein	HPV16-L1 SARS-CoV-2 spike protein/RBD HIV-1 Gag HCV envelope glycoproteins (HCV E1-E2)	HPV SARS-CoV-2 HIV HCV	Murine Human tissue	[42, 98^a^,^100^, ^109^]
GM for: IM prot + viral protein	Whole Lt and/or purified protein	HCV polytope (PT) fused to the N-terminal domain of heat shock protein GP96 (HCV-PT + NT-GP96) (HPV-E7 + NT-GP96) + (HPV-E7 + CT-GP96)	HPV HCV	Murine	[72, 101]
GM for: VLPs	VLPs	HPV late protein 1 (HPV16 L1) HBV small surface antigen (sHBsAg) presenting epitopes derived from the HCV E2 glycoprotein NoV Capsid Prot	HPV HBV HCV NoV	Murine	[102–105]

*GFP* Green fluroescent protein,* GM* genetically modified *Leishmania*,* HBV/HCV* hepatitis B/C virus,* HIV* human immunodeficient virus,* HPV* human papillomavirus, *IM prot* immune-modulating protein,* Lt Leishmania tarentolae, NoV* norovirus,* pLs prot* pathogenic *Leishmania* species protein,* prot* protein,* SARS-CoV-2* severe acute respiratory syndrome coronavirus 2,* VLPs* virus-like particles, * WT* wild type/non-engineered

^a^A purified recombinant protein and *Leishmania* cells expressing a recombinant protein were co-administered

Different protein antigens from pathogenic *Leishmania* species have been investigated in anti-*Leishmania* vaccine research, with *L. tarentolae* used as a vehicle for their production and delivery. For example, administration of *L. tarentolae* engineered to express the A2 antigen from *L. donovani* provided protection against *L. infantum* in a murine model of the infection [77]. Other studies using *L. tarentolae* as a vaccine vehicle exploited the LACK and KMP11 antigens (for the full name of these antigens, see Table 1), that are expressed both in the promastigote and the amastigote stage and are involved in the infection of mammalian hosts [78, 79]. Immunization with *L. tarentolae* expressing these proteins, co-adjuvated with CpG motifs, achieved a reduction of parasite load by *Leishmania major* in a murine model [73]. As a third example, live *L. tarentolae* expressing LPG3 from *L. infantum*, a protein chaperon involved in the biosynthesis of lipophosphoglycans, induced the expression of high level of interferon gamma (IFN-γ) in the murine model, and partial protection against *L. infantum* infection, without co-administration of any adjuvant [80].

As already emphasized, *L. tarentolae* has been exploited not only to express and deliver protein antigens from pathogenic *Leishmania* parasites, but also for the production and delivery of immune-modulating molecules. Recent findings point to CXCL-10 (C-X-C motif chemokine 10) as a potential tool for immunotherapy of cutaneous leishmaniasis, since this molecule favors a shift towards a Th1 pro-inflammatory response [81]. Indeed, inoculation of *L. tarentolae* expressing CXCL-10 in mice inhibited arginase activity and Th2 cytokine expression towards IFN-γ and nitric oxide production [82]. Another example of an immune-modulating molecule investigated in vaccine research is the human neutrophil peptide (HNP1), which is a member of the large antimicrobial peptides (AMPs) group [83]. In a study published in 2017, a strain of *L. tarentolae* engineered for the expression of HPN1 from *L. major* was tested in the murine model; the results showed containment of the parasite load after challenge with *L. major* itself [84].

The contribution of a vector’s saliva in the immunology of leishmaniases has been extensively been studied in recent decades, with identification of highly immunogenic molecules, such as the PpSP15 protein from *Phlebotomus papatasi* [85]. Regarding the use of engineered *Sauroleishmania* in vaccination, the administration of GM *L. tarentolae* expressing the cysteine proteinases A and B (CPA/CPB) genes, in co-administration with a DNA plasmid coding for the sand fly salivary antigen PpSP15, was shown to be very effective in murine models as a vaccine against *L. major* [86]. *Leishmania tarentolae* expressing PpSP15 was also effective in terms of immunization against *L. major* infection when administered in combination with CpG oligodeoxynucleotides [87]. More recently, the combination of two salivary proteins from the sand fly, PpSP15 and PpSP9, co-expressed and delivered by *L. tarentolae*, proved to be effective in immunization against two different *Leishmania* species, *L. major* and *L. tropica* [88].

## Microbial vaccine vehicles*: Leishmania tarentolae* as an anti-viral vaccine

**Fig. 3:**
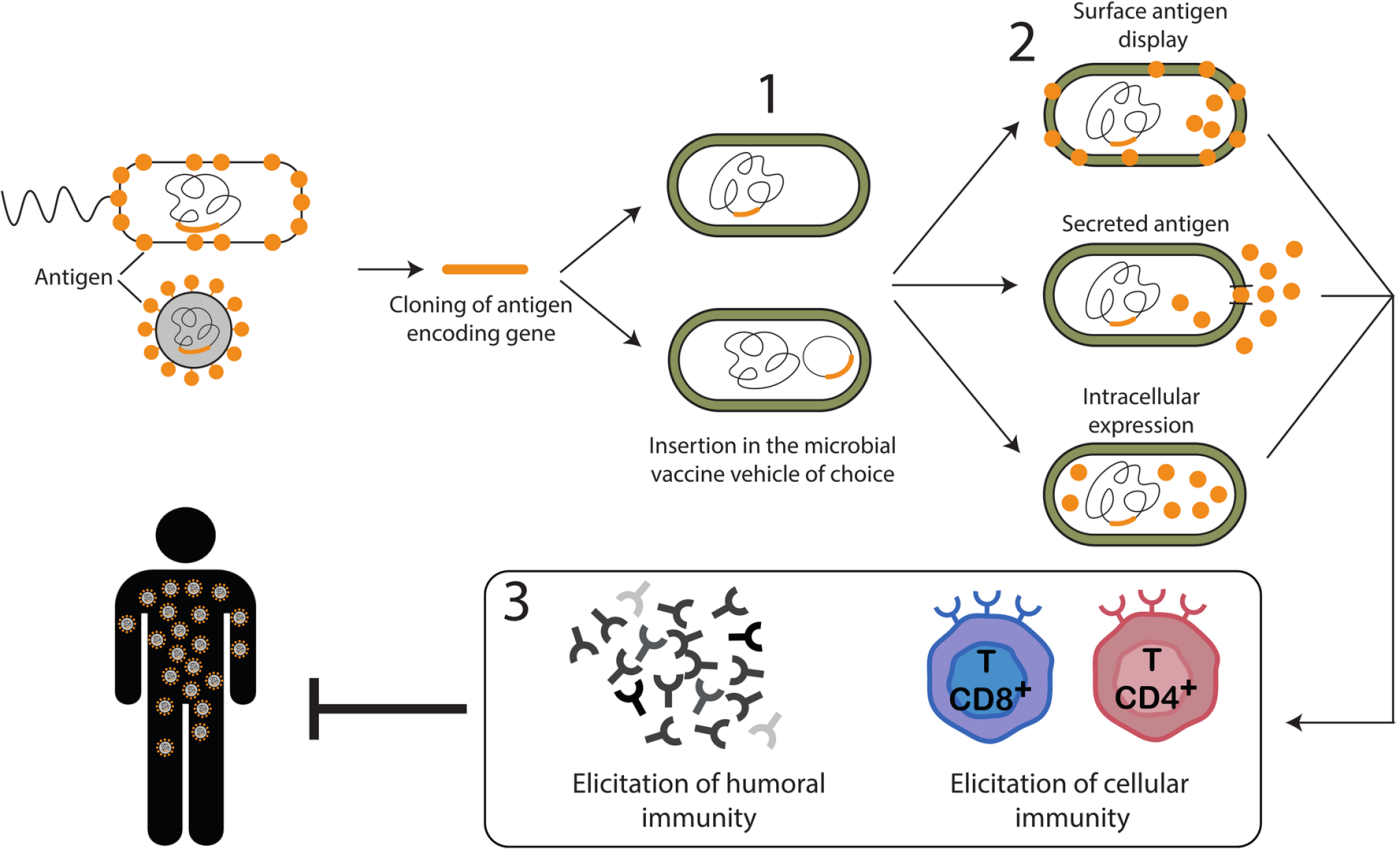
Use of microbial vectors in vaccination against viruses and other pathogens. A gene coding for an antigen is selected from a pathogen and cloned for its expression into a microbial vehicle (*1*).  Different types of expression could be exploited (*2*). The selected microbial vehicle is then used for the immunization of the host (*3*). GM, Genetically modified, WT, wild type

At the time of writing this article, four major vaccine technologies or platforms are being used extensively for the production of antiviral vaccines: (i) RNA vaccines delivered through lipid nanoparticles; (ii) adenovirus-based viral vectors; (iii) subunit vaccines, using recombinant protein antigens, produced in eukaryotic expression systems; and (iv) inactivated or attenuated viruses [89]. In addition to these types of platforms, a fifth approach is based on the administration of whole bacterial cells that are engineered for the expression of viral antigens (Fig. 3) [90–92].

The core idea of this strategy is that GM bacteria can be exploited not only as micro-factories to produce the desired antigen, but also as vehicles for the delivery of the antigens themselves, ideally targeting DCs and lymph nodes. These GM bacteria are referred to as bacterial vaccine vehicles, or as living bacterial vehicles, when used without inactivation. In the context of antiviral vaccination, a major limitation with the use of bacteria as antigen vehicles, or even for the simple production of the antigens, is that protein expressed in bacteria are not subjected to post-translational modifications, which are instead characteristic of proteins produced in mammalian cells [93, 94]. Therefore, viral antigens expressed in bacteria are expected to present structural differences in comparison with antigens produced by mammalian cells during viral infections. In addition to bacteria, yeasts have also been proposed as vaccine vehicles for the production and delivery of viral antigens (see, for example, [95]). Indeed, production of protein antigens in yeasts ensures post-translational modification of the antigen, but the glycosylation pattern in yeast-produced proteins is rather different from that of mammalian proteins [96].

Considering the glycosylation issue, *L. tarentolae* emerges as an alternative solution to produce viral antigens: as outlined above, the glycosylation pattern in this microorganism resembles that of mammals. In addition, both the easy culturing and genetic engineering of *L. tarentolae*, as well as its propensity to target DCs, make this protozoon an ideal candidate for its use as a microbial antigen vehicle for anti-viral vaccination. We emphasize that the use of GM *L. tarentolae* as candidate anti-*Leishmania* vaccines (see above sections) also exploits this microorganism as a vehicle for the antigen. Published studies on the use of *L. tarentolae* as an antigen vehicle in anti-viral vaccination have so far targeted eight different viruses (Table 1). A first study in this area exploited *L. tarentolae* for the expression and delivery of the Gag protein from human immunodeficiency virus type 1 (HIV-1). The assays in the murine model led to an effective immune response, with the production of neutralizing antibodies [42]. Another virus that has been targeted in preclinical studies using GM *L. tarentolae* is human papillomavirus (HPV), in a murine model of the tumor associated with this virus [97]; the results of that study revealed a contained growth of the tumor in vaccinated mice. Finally, GM *L. tarentolae* expressing the spike protein of SARS-CoV-2 has recently been assayed in mice, and the production of neutralizing antibodies was observed [98]. In the latter study, rectal administration of the engineered strain of *L. tarentolae* was also shown to be effective in the induction of the neutralizing response, paving the way toward the use of *L. tarentolae* as an antigen vehicle for mucosal vaccination.

In addition to the exploitation of *L. tarentolae* as an antigen vehicle, this protozoon has also been used to produce viral protein antigens that have been assayed as purified proteins, alone or in combination with GM *L. tarentolae* strains [e.g. 98], or virus-like particles, that have been assayed in preclinical studies against four different viruses (Table 1). Finally, *L. tarentolae* has also been investigated for the production of a candidate vaccine against the apicomplexan protozoon *Toxoplasma gondii* [99]; in that study, a multi-epitope vector-based vaccine, based on immunodominant epitopes of five *Toxoplasma* antigens, conferred protective immunity against acute toxoplasmosis in BALB/c mice [99].

## Concluding remarks

*Leishmania tarentolae* has a great potential as a candidate vaccine, both as non-engineered, WT organism, possibly in combination with adjuvating molecules favoring Th1-biased responses, or after its engineering, for the expression of antigens and/or immune-modulating molecules. However, several research issues still need to be addressed for an effective translation into practice. The use of *L. tarentolae* as a wild-type whole organism for vaccination of dogs (and possibly humans) will require further studies in rodent models, with also the need to understand whether the parasite can actually replicate within the mammalian host, ensuring adequate stimulation of the immune response, without causing any pathological alteration. In this context, a very important issue is whether wild-type *L. tarentolae* administered as living cells would benefit from co-administration with an adjuvant. Indeed, co-administration with adjuvants could stimulate an excessive immune response against *L. tarentolae*, with early inactivation of the parasites, and thus reduced replication. Therefore, investigations on adjuvating strategies capable of favoring the immune response in the Th1 direction while allowing *L. tarentolae* to undergo a few replication cycles will be of great importance. Regarding the strains of *L. tarentolae* engineered for vaccination against pathogenic protozoa of the genus *Leishmania*, viruses or other infectious agents, the effective use of these strains will require addressing the problems related with their status as GM organisms, obtained by incorporating one or more genes for antibiotic resistance. The actual use of these strains could therefore require the removal of resistance genes or the development of metabolic complementation strategies for the selection of transformed strains as an alternative to antibiotic-based selection. More generally, the production of *L. tarentolae* cells for an effective use in vaccination campaigns will require production to be scaled up to the level of industrial bio-fermenters, with an optimization of the culture media, in the absence of antibiotic pressure (see above). In any case, the increased interest in *L. tarentolae*, both as a vaccine platform and more generally as a micro-factory to produce recombinant proteins for a variety of applications, will likely lead to an acceleration of research on this organism and to solutions to the above issues.

## Data Availability

Not applicable.
